# Hepatitis B virus (HBV) screening, linkage and retention-in-care in inclusion health populations: Evaluation of an outreach screening programme in London

**DOI:** 10.1016/j.jinf.2023.12.012

**Published:** 2023-12-29

**Authors:** Emily Martyn, Sive O’Regan, Philippa Harris, Mark Leonard, Martha Veitch, Binta Sultan, Philippa C. Matthews, Indrajit Ghosh, Alistair Story, Julian Surey

**Affiliations:** 1 London School of Hygiene & Tropical Medicine, Keppel St, London, WC1E 7HT, UK; 2 The Francis Crick Institute, 1 Midland Road, London NW1 1AT, UK; 3 Find & Treat Service, Division of Infection, University College London Hospitals NHS Foundation Trust, 250 Euston Road, London, NW1 2BU, UK; 4 Mortimer Market Centre, Central and North London NHS Foundation Trust, Capper Street, London, WC1E 6JB, UK; 5 Division of Infection and Immunity, University College London, Gower St, London, UK, WC1E 6BT; 6 Department of Infectious Diseases, University College London Hospitals NHS Foundation Trust, 250 Euston Road, London, NW1 2BU, UK; 7 Collaborative Centre for Inclusion Health, University College London, Gower St, London, UK, WC1E 6BT; 8 Institute of Global Health, University College London, Gower St, London, UK, WC1E 6BT; 9 Universidad Autonoma de Madrid, Ciudad Universitaria de Cantoblanco, 28049 Madrid, Spain

**Keywords:** Hepatitis, Hepatitis B, Inclusion Health, Key Populations, Outreach, Screening

## Abstract

**Objectives:**

We evaluate a hepatitis B virus (HBV) screening programme, delivered by a specialist pan-London multidisciplinary outreach team, to understand population characteristics and care cascade among people who experience extreme social exclusion (Inclusion Health (IH) groups).

**Methods:**

Point-of-care HBV screening was performed in temporary accommodation for people experiencing homelessness (PEH) and people seeking asylum (initial accommodation centres, IACs) via a mobile unit staffed by peers with lived experience, nurses, and doctors. We analysed demographics and HBV characteristics of adults screened between May 2020 and January 2022. We ascertained linkage-to-care (LTC), retention-in-care (RIC) and loss-to-follow-up (LTFU). People LTFU were contacted by peers to re-engage in care.

**Results:**

2473 people were screened: 809 in IACs, 1664 in other temporary accommodation. Overall hepatitis B surface antigen (HBsAg) prevalence was 1.7% (43/2473), highest in IACs (3.5%, 28/809). LTC within 3 months was 56% (24/43) and RIC, 87% (26/30). LTC was higher when referred to a local IH-specialist hepatitis service, compared to other services (77%, 17/22 vs 33%, 7/21; p=0.006). LTFU was 30% (13/43), reduced to 21% (9/43) after intervention by peers.

**Conclusion:**

Our findings support outreach screening among IH populations and peer-supported linkage to IH-specialist hepatitis services. We recommend increased HBV testing and HBV-specific IH specialist services.

## Introduction

Hepatitis B virus (HBV) is responsible for an estimated 296 million chronic infections globally. Despite an effective vaccine and availability of suppressive antiviral treatment, there are still approximately 1.5 million new infections, and 0.8 million deaths each year due to viral hepatitis related cirrhosis and hepatocellular carcinoma (HCC) (1). The World Health Organization (WHO) set an ambitious target to eliminate viral hepatitis as a public health threat by 2030 in line with the United Nations Sustainable Development Goals (2).

Approximately 10% of people living with chronic hepatitis B (CHB) are aware of their diagnosis, and linkage-to-care remains low, even in high-income countries (3, 4). Estimates suggest, despite 20% of people living with CHB globally are eligible for treatment, only 2% were on treatment in 2019 (5, 6). Even though progress is being made, 2020 modelling data suggests that current elimination targets will not be reached until >2051(4, 6).

To achieve elimination goals, greater efforts are needed to diagnose and treat people living with chronic HBV. Inclusion Health (IH) populations are recognised as important groups in the mission to eliminate hepatitis, since they are disproportionately affected by HBV and yet experience extreme social exclusion and barriers to accessing healthcare (7). These groups include, but are not limited to, people experiencing homelessness (PEH), commercial sex workers, incarcerated individuals, people who inject drugs (PWID) and people seeking asylum (8). In July 2022, the WHO published its first guideline to specifically targeting equitable, accessible and acceptable services for HIV, viral hepatitis and STI prevention among these groups, also termed “Key Populations”, recognising this strategy as critical to achieving global hepatitis elimination goals (9). In the UK, there is a specific call from public health agencies to focus screening on population groups in which there is increased risk of infection, with specific recognition of the need for enhanced attention on vulnerable migrants (10).

The “Find&Treat” (F&T) service in London is dedicated to reaching IH populations. It is a peer-centred specialist multidisciplinary outreach team that works in conjunction with over 200 NHS and third sector front line services to improve access to healthcare and tackle infectious diseases. A key feature of F&T is the integration of peer support workers (people with lived experience) as key members of the healthcare team in an ‘assistant practitioner’ role. Peers are trained to lead in active case finding, blood-borne virus (BBV) screening using point-of-care-tests, transient elastography to assess liver fibrosis, and referral and linkage to secondary care. Peers are provided with support for professional development and remunerated on the same scale as NHS non-clinical roles. This model of peer-led support has been shown to be effective at improving engagement in care, for example in marginalised populations with chronic hepatitis C virus (HCV) infection (11, 12).

We set out to evaluate an opportunistic HBV screening programme of IH populations conducted by F&T in London. We describe this subset of IH populations living with HBV infection and determine their linkage to, and retention in, secondary care. Based on our findings, we suggest improvements for HBV IH services.

## Methods

Between May 2020 and January 2022, peers with lived experience, specialist nurses and doctors offered on-site BBV point-of-care testing (POCT) to adults (>18 years) living at hostels for PEH, Covid hotels (emergency UK Home Office funded temporary accommodation for PEH during the Covid-19 pandemic) and initial accommodation centres (IACs, temporary accommodation for people seeking asylum) across London.

Individuals were given brief counselling about BBVs and then offered screening. A questionnaire was administered by peers, collecting information on age, gender, country of birth, first language, sexual orientation, drug and alcohol use, HBV vaccination status and knowledge of HBV status (supplementary figure 1). On-site translators offered by third sector organisations or telephone translation services were used to facilitate communication (13).

POCT for hepatitis B surface antigen (HBsAg) (HBsAg test, Turklab, Izmir, Turkey) and combined HIV antibody/antigen and syphilis antibody testing (INSTI Multiplex HIV/Syphilis test, BioLytical Laboratories, British Columbia, Canada) were undertaken using finger prick blood sampling. Anti-hepatitis C virus (HCV) antibody was tested using rapid POCT (OraQuick anti-HCV test, OraSure Technologies, Pennsylvania, USA). In the event of a positive HBsAg test, where possible, on-site assessment of liver fibrosis was undertaken by trained peers or nurses, using transient elastography measured by FibroScan (FibroScan® Mini+ 430, FibroScan® M+ Probe, Echosens, France) and blood tests. The median of at least ten liver stiffness measurements was taken, with an interquartile range of <30% for a valid, high quality reading. Fibrosis was staged as follows: F0/1 (no/minimal fibrosis) <7.2kPa, F2 (moderate fibrosis) 7.3 - 9.4 kPa, F3 (advanced fibrosis) 9.5 - 12.2 kPa and F4 (cirrhosis) >12.2 kPa (14). Questionnaires and test results were uploaded the same day to a secure electronic, password encrypted database (Microsoft Access, Washington, United States).

A referral was then made to either an inclusion health focused local specialist hepatitis centre (Mortimer Market Centre, Central and North West London NHS trust) or to other local hepatitis services. The Mortimer Market Centre service has strong communication links with the F&T service, offers flexible clinics and has expertise in looking after patients experiencing multi-disadvantage. If it was inconvenient for the patient to travel to Mortimer Market Centre, referral was made to other specialist hepatitis services across London, based on the client’s location preference. Financial and physical support to attend specialist appointments was offered by peers from the F&T team and third sector organisations (13, 15). Decision to initiate treatment was undertaken in specialist hepatitis services based on national treatment guidelines (16) (See Appendix 2).

In October 2022, we reviewed local electronic patient records and contacted external referral specialist centres to collect information on HBV investigations, treatment status and establish linkage-to-care (LTC), retention-in-care (RIC) and loss-to-follow-up (LTFU). Definitions to define LTC, RIC and LTFU are not well established for HBV, therefore we used the following pragmatic definitions:

1. LTC: assessment by a specialist within 3 months of diagnosis. This time frame was based on literature from the HIV field (17).
2. RIC: more than one appointment in specialist services between screening and end of follow-up period (October 2022)
3. LTFU: never seen in secondary care at any point between screening test and end of follow up (October 2022)

Anyone who was identified as LTFU was contacted by the F&T team and a multi-step approach was used to link them back into care: an attempt to contact the person directly via phone/text, then contacting the last known accommodation or key worker, then checking NHS wide databases for their most recent GP details, if they gave consent for GP contact. We re-assessed in March 2023 to assess whether any of those LTFU were re-engaged in care.

Specialist referral services were contacted and asked if the following tests were performed (based on recommendations by in guidelines from National Institute for Health and Care Excellence (NICE) for HBV work-up (16):

- Hepatitis B e antigen/antibody
- HBV DNA viral load
- Anti-HCV antibody
- Hepatitis delta virus (HDV) antibody
- HIV antibody
- Alanine transaminase (ALT) (upper limit of normal >19 IU/L females, >30 IU/L males)
- Platelets
- Liver ultrasound, alpha-fetoprotein (aFP) testing*
- Quantitative HBsAg
- Transient elastography (TE)

*Six monthly HCC surveillance with ultrasound and serum aFP measurement is recommended in anyone with ≥F2 fibrosis or cirrhosis, and considered if they are over 40 years, have a family history of HCC, or HBV viral load ≥20 000 IU/ml (16).

We calculated the Fibrosis-4 (FIB-4) score, a non-invasive fibrosis score, using the following formula:

- FIB-4 = (Age in years x AST) / (Platelet count * √ALT)

Cut-offs for the Fib4 score are as follows: <1.45 Fibrosis stage 0-1, 1.45-3.5 Fibrosis stage 2-3, >3.25 Fibrosis stage 4-6 (18).

Analysis of baseline characteristics was undertaken using R Studio (4.2.0). Comparison of LTC, RIC and LTFU in different groups was analysed in GraphPad (9.5.1) using Fisher’s Exact Test.

### Governance and ethical approvals

Verbal and written consent was given by clients to share data for ongoing referral and anonymously for service evaluation and research. This is a service evaluation and therefore according to the NHS Health Research Authority decision tool did not require ethical approval (19). It is registered with the University College London Infection Division Quality Improvement Governance Department.

## Results

### Overall population characteristics

Between May 2020 and January 2022, 2473 people from IH populations in London were screened for HBV by F&T: 809 in IACs and 1664 in other temporary accommodation (e.g. Covid hotels, hostels for PEH). The median age was 40 (IQR 30,50 and 74 % (1840/2473) were male). Those in IACs were people seeking asylum, whereas those in the other temporary accommodation were majority PEH.

Overall prevalence of HBsAg positivity was 1.7% (43/2473). HBsAg prevalence in IACs was 3.5% (28/809) and those in the remaining sites had a HBsAg prevalence of 0.9% (15/1664). The median age of people living with HBV (HBsAg+) was 33 years (IQR 24, 42), lower than those with an unreactive HBsAg (40, IQR 30, 51) (p=0.0008) (Table 1). Of those who screened positive for HBsAg, 49% (21/43) were born in Africa, 33% (14/43) in Europe and 16% (7/43) in Asia. There were 18 different first languages spoken. The majority never injected drugs (81%, 35/43), never drank alcohol (65%, 28/43) and identified as heterosexual (79%, 34/43). There were very few co-infections (<5 HCV, HDV and HIV, exact number not given to reduce risk of deductive disclosure, table 1 & 2).

### HBV characteristics

Of all cases where HBV was identified, it was a new a new diagnosis in 67% (29/43) (table 2). 63% (27/43) were Hepatitis B e-Antigen (HBeAg) negative, 56% (24/43) Anti-HBe-Antibody positive, 28% (12/43) had HBV DNA >2000 IU/ml, and 44% (19/43) had qHBsAg > 1000 IU/ml. Notably, investigations revealed evidence of liver inflammation and/or fibrosis: 35% (15/43) had elevated ALT, 12% (5/43) had F3/4 fibrosis on transient elastography, 16% (7/43) had a Fib4 score between 1.45 and 3.25 indicating fibrosis stage 2-3, and 12% (5/43) had coarsened liver echotexture on ultrasound, indicating possible cirrhosis. 12% (5/43) were recommended to start nucleoside analogue (NA) therapy following review in hepatitis clinic.

Of those ever seen in specialist care, 86% (26/30) had qHBsAg measured, 83% (25/30) had anti-HDV serology recorded, and all had anti-HCV and HIV tests. Of those seen in specialist care and meeting NICE guidelines for increased HCC surveillance, 60% (6/10) had an ultrasound scan and 70% (7/10) had aFP measured (16). No cases of HCC were detected.

### Care cascade

**Figure 1:**
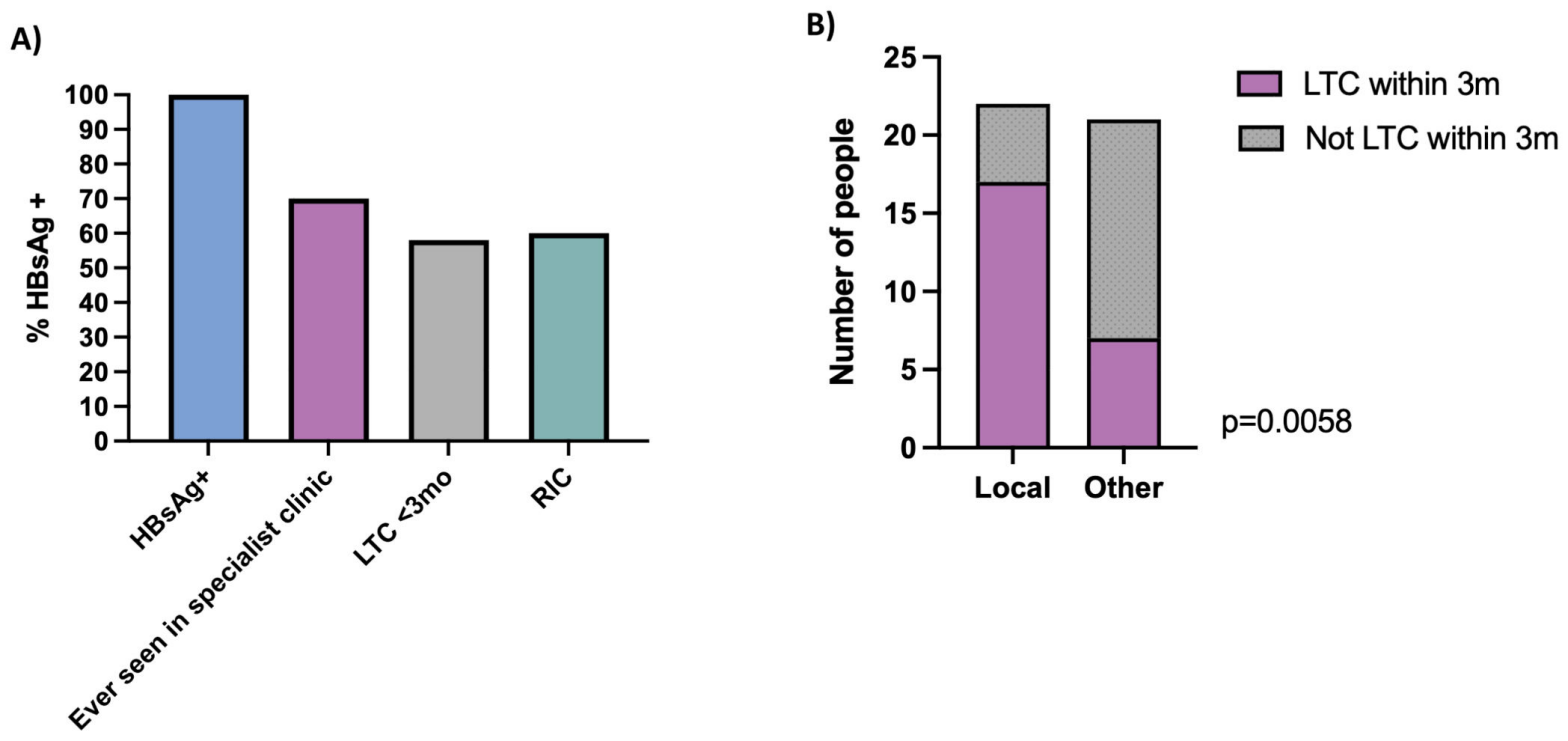
Follow-up outcomes in a population of 43 adults who screened positive on point-of-care testing for hepatitis B surface antigen (HBsAg) between May 2020 - January 2022 in London, in emergency accommodation for adults experiencing homelessness and seeking asylum. (A) Hepatitis B Care Cascade; (B) Linkage-to-care by referral site; Local: referral to Find & Treat local hepatitis services; Other: referral to other hepatitis services across London. Compared using Fisher’s Exact Test. Abbreviations: HBsAg+, person screening positive for point-of-care testing for HBsAg; Ever seen in specialist clinic, seen at some time between screening positive and October 2022; LTC <3mo, linked to care within 3 months of positive screening test; RIC, retained in care (>1 appointment in secondary care).

The overall care cascade in October 2022 is illustrated in figure 1A. 56% (24/43) were LTC within 3 months. More people residing at IACs compared to PEH were LTC within 3 months, although this did not reach significance (68%, 19/28, vs 33%, 5/15; p=0.052). 70% (30/43) attended an appointment in a specialist clinic at some point during the follow up period. Among those who attended a specialist clinic, 87% (26/30) had at least one further specialist appointment (i.e. RIC), which did not differ between people diagnosed in IACs compared to PEH (86%, 19/22 vs 88%, 7/8, p=0.99). LTFU was 30% (13/43) in October 2022, and was greater among PEH than IACs, although not statistically significant (47%, 7/15 vs 21% 6/28, p = 0.16).

### Impact of specialist IH services

LTC was significantly higher among those referred to local specialist IH hepatitis services, compared to those referred to other hepatitis clinics across London (77%, 17/22 vs 33%, 7/21; p=0.006) (figure 1B). Of people who were ever seen in specialist care, 90% (19/20) who were seen in the local inclusion health sensitive specialist hepatitis services were RIC, compared to 70% (7/10) of those seen in other hepatitis clinics, but this difference was not significant (p=0.09). Significantly fewer people referred to the local specialist hepatitis services were LTFU compared to those referred to other services (9%, 2/22 vs 52%, 11/21, p=0.003).

By March 2023, 31% (4/13) of those identified as LTFU and four people who had not been seen in ≥ 1 year (4/12, 33%) were re-engaged with hepatitis follow-up by specialist peers.

## Discussion

We describe an HBV screening programme targeting underserved communities, with a focus on IH populations, including people living in temporary accommodation during the Covid-19 pandemic. Our main findings and recommendations from this service evaluation are summarised in Table 3.

Most people screened were seeking asylum (temporarily housed in IACs), and PEH in temporary Covid accommodation and hostels. Total HBsAg positivity was 1.7%: but this was higher, at 3.5%, among people seeking asylum. UKHSA estimates the overall population prevalence is 0.45% (95% CI 0.35% to 0.60%). This is lower when estimated using primary care data (0.05%), suggesting that many people living with HBV may not be registered with their GP and highlights a gap in diagnosis (10, 20). Our data point towards an increased prevalence of HBV in IH populations compared to the general population, supporting the hypothesis that some of those undiagnosed may be in IH populations.

In a drive to reach hepatitis elimination goals, NHS England have launched a pilot project screening all people attending Emergency Departments for HIV, HCV and HBV (21). This is particularly relevant to IH populations who are more likely to use Emergency Departments to access healthcare (22). As new cases of chronic HBV infection are diagnosed, understanding IH engagement in hepatitis care is becoming even more important. There is increasing recognition from global organisations that elimination of hepatitis will require targeted services for IH or “key” populations, providing equitable access to healthcare for stigmatised and marginalised populations (9, 10).

To our knowledge, this is the only study reporting specifically on the care cascade for chronic HBV in PEH. In comparison to other published data on refugees and migrants, our reported rates of LTC and RIC are high, with low LTFU; a study of newly settled refugees in the United States found 6 month linkage to care was 36% and another study including refugees and asylum seekers in southern Italy found that only 43% were RIC at one year (23, 24). However, comparison is limited since definitions of LTC, RIC and LTFU are variable (25, 26). It will be important to reach consensus definitions to improve our understanding of published data, compare efficacy of interventions and to provide guidance for health services on the gold standard in CHB care.

Asian ethnicity, proximity of residence to clinic, older age and being on treatment have been identified as predictors of RIC, whereas refugee status predicts LTFU (26, 27). Accessible care, patient and provider education and reduction in stigma are key factors to improve the HBV care cascade in the United States (28).

In this evaluation, we found that referral to a specialist inclusion health hepatitis service resulted in significantly higher rates of LTC and lower LTFU compared to referral to other hepatitis services. This may be due to easier communication between F&T and the local specialist hepatitis service and more established referral pathways. This also reflects local expertise in facilitating care for IH populations, for example, flexible appointments and ensuring use of translators.

A key component of F&T’s success is peer support. In this evaluation, expert peers with lived experience re-engaged 33% of those LTFU and another 33% of people who hadn’t been reviewed in specialist care for a year or longer. Peer support has a track record of improving engagement in care in many conditions, including HCV and HIV (11, 29) and our local hepatitis services are the first in the UK to secure funding for a specialist HBV peer. However, people living with HBV are diverse, as illustrated in this study with PEH and people seeking asylum. Therefore, investment is needed into recruiting peers with lived experience to support the diverse HBV population.

Digital solutions are sometimes useful in other infectious diseases, for example, a smartphone app improved LTC in HIV (30). Innovative use of technology should be leveraged to assist health services addressing the needs of IH populations living with HBV.

There are several limitations of this service evaluation. Firstly, the number of people living with HBV was small and screening was carried out in London, therefore may not reflect IH populations in other locations and cities. The initial screening programme began during the Covid-19 pandemic and referral pathways may have been impacted by the strain on the NHS at this time. Most people living with HBV were found in IACs and the care cascade will be heavily impacted by the current government policies on people seeking asylum. There was a high proportion of unknown hepatitis delta serology. Some results came from external referral clinics and therefore we cannot conclude that the absence of hepatitis D result is due to lack of testing, or result omission. Hepatitis D is globally underdiagnosed and we aim to address this in future evaluations (31). Due to small numbers, we were unable to understand the extent of liver disease in particular subgroups, such as HCV or HDV coinfection. We did not address vaccination status, a critical tool in prevention of HBV, and this would be an essential component of further work and service development.

In conclusion, we describe an effort to screen for HBV, link and maintain retention in HBV care among IH populations in London. Although modest in size, these are important findings which may help inform services targeting underserved groups, forming an essential step to achieving global HBV elimination goals.

## Supplementary Material

Supplementary file

## Figures and Tables

**Table 1:** Population demographics by HBsAg status.

Demographic	HBsAg positive (n=43)	HBsAg negative (n=2430)
**Age (median [IQR])**	32.50 [24.25, 42.00]	40.00 [30.00, 51.00]
**Age group, n (%)**		
18-30	16(37)	573 (24)
31-40	12(28)	599 (25)
41-50	10 (23)	582 (24)
51-60	<5*	400 (16)
60+	<5*	193 (7.9)
Unknown	<5*	83 (3.4)
**Gender, n (%)**		
Female	5 (12)	558 (23)
Male	37(86)	1803 (74)
Trans female	0	<5*
Trans male	0	-*
Unknown	1 (2.3)	61 (2.8)
**Site Type, n (%)**		
Other temporary accommodation^θ^	15(35)	1649 (68)
Initial accommodation centres	28 (65)	781 (32)
**Continent of birth, n (%)**		
Africa	21 (49)	510 (21)
Asia	7 (16)	451 (19)
Australia	0 (0.0)	6 (0.2)
Europe	14 (33)	1177(48)
North America	0	49 (2.0)
South America	0	102 (4.2)
Unknown	1 (2.3)	135 (5.6)
**Intravenous drug use, n (%)**			
Ever	<5*	357 (15)	
Never	35 (81)	1961 (81)	
Unknown	<5*	112 (4.6)	
**Alcohol, n (%)**			
Daily	<5*	347 (14)	
Less than daily	8 (19)	563 (23)	
Never	28 (65)	1212 (50)	
Unknown	<5*	308 (13)	
**Sexual orientation, n (%)**			
Heterosexual	34 (79)	2021 (83)	
Bisexual	<5*	66 (2.7)	
Gay / Lesbian	<5*	42 (1.7)	
Other	0	25 (1.0)	
Unknown	7 (16)	276 (11)	
**Hepatitis C Antibody, n (%)**			
Reactive	<5*	222 (9.1)	
Unreactive	35 (81)	2065 (85)	
Indeterminate	0	<5*	
Unknown	<5*	-*	
**Hepatitis C RNA, n (%)**			
Reactive	<5*	76 (3.1)	
Unreactive	<5*	133 (5.4)	
Unknown	<5*	13 (0.5)	
**HIV Antibody n (%)**			
Indeterminate	0	<5*	
Reactive	<5*	24 (1.0)	
Unreactive	40 (93)	2271 (93)	
Unknown	<5*	135 (5.6)	

Abbreviations: HBsAg, Hepatitis B Surface Antigen; RNA, Ribonucleic Acid; HIV, Human Immunodeficiency Virus. *exact number not given to mitigate risk of deductive disclosure; ^θ^Other temporary accommodation: Covid Hotels, Hostels, Home visits and other locations (explained in text), grouped to mitigate risk of deductive disclosure.

**Table 2:** Hepatitis B characteristics in the inclusion health population.

Hepatitis B Characteristic	Overall (43)
**HBV previously known n (%)**	
No	29(67)
Yes	10 (23)
Unknown	4 (9.3)
**e-Antigen n (%)**	
Negative	27(63)
Positive	3 (7.0)
Unknown	13 (30)
**Anti-e antibody n (%)**	
Negative	4 (9.3)
Positive	24 (56)
Unknown	15(35)
**Hepatitis B DNA (IU/ml) n (%)**	
<2000	19(44)
2000-19999	8 (18)
>20000	4 (9.3)
Unknown	12 (28)
**Quantitative Hepatitis B surface Antigen (IU/ml) n (%)**	
<1000	3 (7.0)
>1000	19 (44)
Unknown	21 (49)
**Abnormal ALT n (%)**	
No	15(35)
Yes	15 (35)
Unknown	13 (30)
**Transient Elastography, Metavir fibrosis score n (%)**	
F0/1	19 (44)
F2	3 (7.0)
F3	3 (7.0)
F4	2 (4.7)
Unknown	16(37)
**Fib4 score n (%)**	
<1.45	14 (33)
1.45-3.25	7 (16)
Unknown	22 (51)
**Ultrasound Scan result n (%)**	
Normal Liver	13 (30)
Coarsened liver echotexture	5 (12)
Steatosis	3 (7.0)
Unknown	22 (51)
**Management n (%)**	
Monitoring	25 (58)
Nucleos(t)ide analogue	5 (12)
Unknown	13 (30)
**Hepatitis Delta Antibody n (%)**	
Negative	-*
Positive	<5*
Unknown	18(42)

ALT = Alanine transaminase. Abnormal = above the upper limit of normal (19 IU/L Females; 30 IU/L Males). *exact number not given to mitigate risk of deductive disclosure. Transient elastography cutoffs F4 >12.2, F3 9.4-12.1, F2 7.2 – 9.3, F0/1 (Li et al., 2016). Fib4 score: <1.45 Fibrosis stage 0-1, 1.45-3.5 Fibrosis stage 2-3, >3.25 Fibrosis stage 4-6 (18).

**Table 3:** Future recommendations for Inclusion Health Chronic Hepatitis B services following this service evaluation.

Finding	Recommendation
3.5% HBsAg prevalence in lACs.	Targeted public health interventions e.g., screening, vaccination, and education programmes.
0.9% HBsAg prevalence in PEH (higher than baseline UK prevalence estimate).	Further evaluation of HBV screening is needed in this population.
18 different first languages spoken.	Support and fund access to translators, ideally on-site but also via telephone.
Significantly higher LTC for those seen in local specialist services compared to those referred to other hepatitis services.	Develop robust referral pathways to specialist clinics. Strengthen links between specialist hepatitis services and outreach services across London. Improve specialist clinics awareness of IH clients, enable flexible clinics, avoid automatic discharge. Use IH outreach team support if multiple DNAs and uncontactable.
Incomplete HBV work up in those LTC.	Make each health encounter count: ˚ Point of care testing for extended HBV testing e.g., HBV viral load, transient elastography ˚ Finger prick small volume blood testing to allow self-testing or assisted testing by peers who are not trained in phlebotomy.
31% of those who were LTFU and 33% of those not seen for ≥ 1 year, were re-engaged in care by peers.	Support and invest in peer support workers. Invest in qualitative studies with different communities to understand unmet need. Decentralised care to improve access e.g., outreach community clinics, Find & Treat mobile eBike clinic.

Abbreviations: HBsAg, Hepatitis B Surface Antigen; IAC, Initial Accommodation Centre; PEH, People Experiencing Homelessness; HBV, hepatitis B virus; LTC, Linkage-to-care

## Highlights

- Hepatitis B disproportionately affects people experiencing extreme social exclusion
- Specialist services enable people experiencing exclusion to attend hepatitis clinic
- Peers with lived experience can help re-engage people in hepatitis B care
