# Supplementary material for: Hepatitis B virus (HBV) screening, linkage and retention-in-care in inclusion health populations: Evaluation of an outreach screening programme in London
Source: J Infect. Author manuscript; Available in PMC 2024 Feb 29. (PMC7615690; doi:10.1016/j.jinf.2023.12.012)
Supplement: Supplementary file [file EMS194258-supplement-Supplementary_file.pptx]

## Slide 1
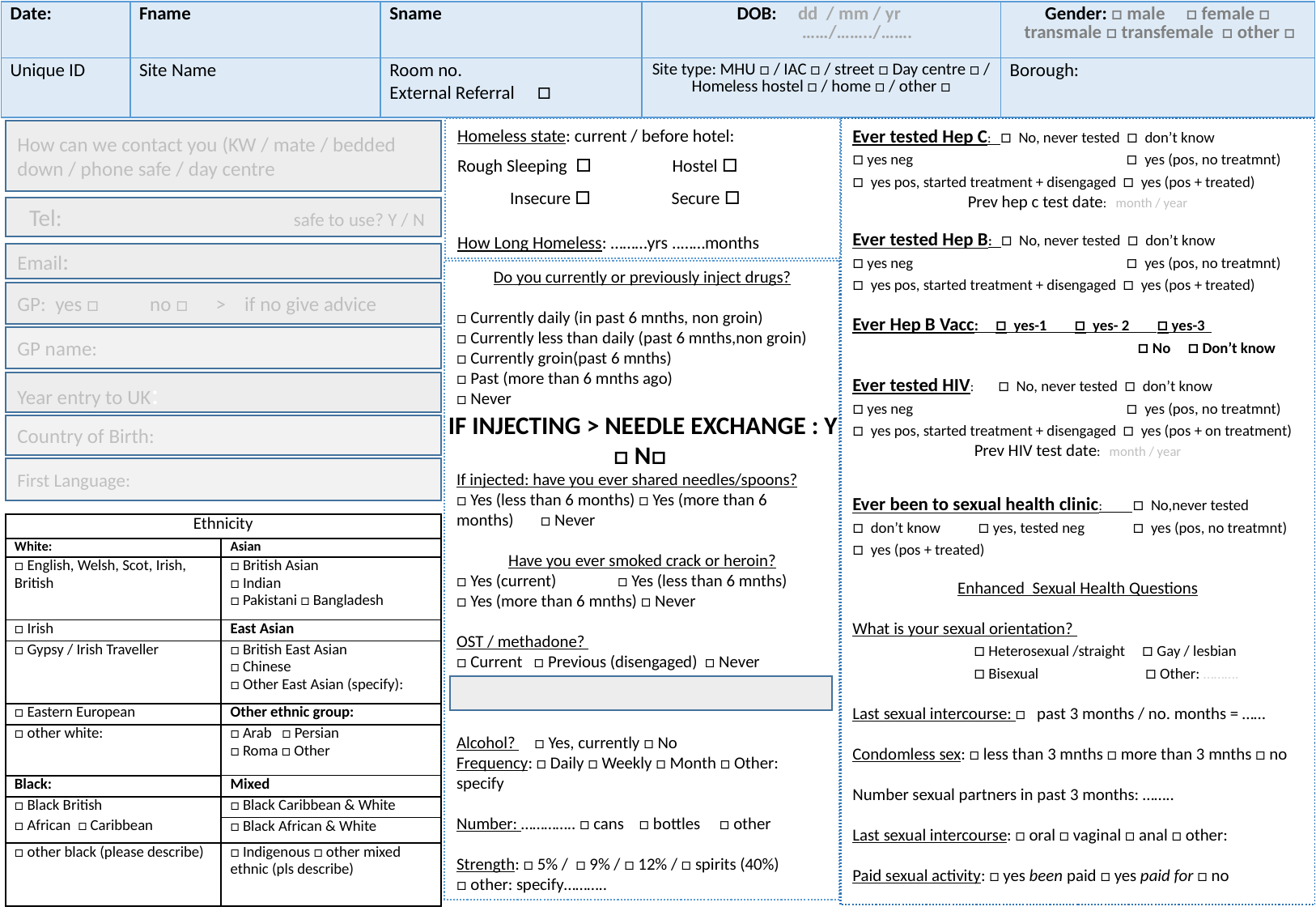

| Date: | Fname | Sname | DOB: dd / mm / yr ……/……../……. | Gender: □ male □ female □ transmale □ transfemale □ other □ |
| --- | --- | --- | --- | --- |
| Unique ID | Site Name | Room no. External Referral □ | Site type: MHU □ / IAC □ / street □ Day centre □ / Homeless hostel □ / home □ / other □ | Borough: |
Homeless state: current / before hotel:
Rough Sleeping □ Hostel □
 Insecure □ Secure □
How Long Homeless: ………yrs ..……months
Ever tested Hep C: □ No, never tested □ don’t know
□ yes neg □ yes (pos, no treatmnt)
□ yes pos, started treatment + disengaged □ yes (pos + treated)
Prev hep c test date: month / year
Ever tested Hep B: □ No, never tested □ don’t know
□ yes neg □ yes (pos, no treatmnt)
□ yes pos, started treatment + disengaged □ yes (pos + treated)
Ever Hep B Vacc: □ yes-1 □ yes- 2 □ yes-3
		 □ No □ Don’t know
Ever tested HIV: □ No, never tested □ don’t know
□ yes neg □ yes (pos, no treatmnt)
□ yes pos, started treatment + disengaged □ yes (pos + on treatment)
Prev HIV test date: month / year
Ever been to sexual health clinic: □ No,never tested
□ don’t know □ yes, tested neg □ yes (pos, no treatmnt)
□ yes (pos + treated)
Enhanced Sexual Health Questions
What is your sexual orientation?
	□ Heterosexual /straight □ Gay / lesbian
	□ Bisexual □ Other: ……….
Last sexual intercourse: □ past 3 months / no. months = ……
Condomless sex: □ less than 3 mnths □ more than 3 mnths □ no
Number sexual partners in past 3 months: ……..
Last sexual intercourse: □ oral □ vaginal □ anal □ other:
Paid sexual activity: □ yes been paid □ yes paid for □ no
How can we contact you (KW / mate / bedded down / phone safe / day centre
Tel: safe to use? Y / N
Email:
Do you currently or previously inject drugs?
□ Currently daily (in past 6 mnths, non groin)
□ Currently less than daily (past 6 mnths,non groin)
□ Currently groin(past 6 mnths)
□ Past (more than 6 mnths ago)
□ Never
If injected: have you ever shared needles/spoons?
□ Yes (less than 6 months) □ Yes (more than 6 months) □ Never
Have you ever smoked crack or heroin?
□ Yes (current) □ Yes (less than 6 mnths)
□ Yes (more than 6 mnths) □ Never
OST / methadone?
□ Current □ Previous (disengaged) □ Never
OST prescriber
Alcohol? □ Yes, currently □ No
Frequency: □ Daily □ Weekly □ Month □ Other: specify
Number: ………….. □ cans □ bottles □ other
Strength: □ 5% / □ 9% / □ 12% / □ spirits (40%)
□ other: specify………..
GP: yes □ no □ > if no give advice
GP name:
Year entry to UK:
IF INJECTING > NEEDLE EXCHANGE : Y □ N□
Country of Birth:
First Language:
| Ethnicity | |
| --- | --- |
| White: | Asian |
| □ English, Welsh, Scot, Irish, British | □ British Asian □ Indian □ Pakistani □ Bangladesh |
| □ Irish | East Asian |
| □ Gypsy / Irish Traveller | □ British East Asian □ Chinese □ Other East Asian (specify): |
| □ Eastern European | Other ethnic group: |
| □ other white: | □ Arab □ Persian □ Roma □ Other |
| Black: | Mixed |
| □ Black British □ African □ Caribbean | □ Black Caribbean & White |
| | □ Black African & White |
| □ other black (please describe) | □ Indigenous □ other mixed ethnic (pls describe) |

## Slide 2
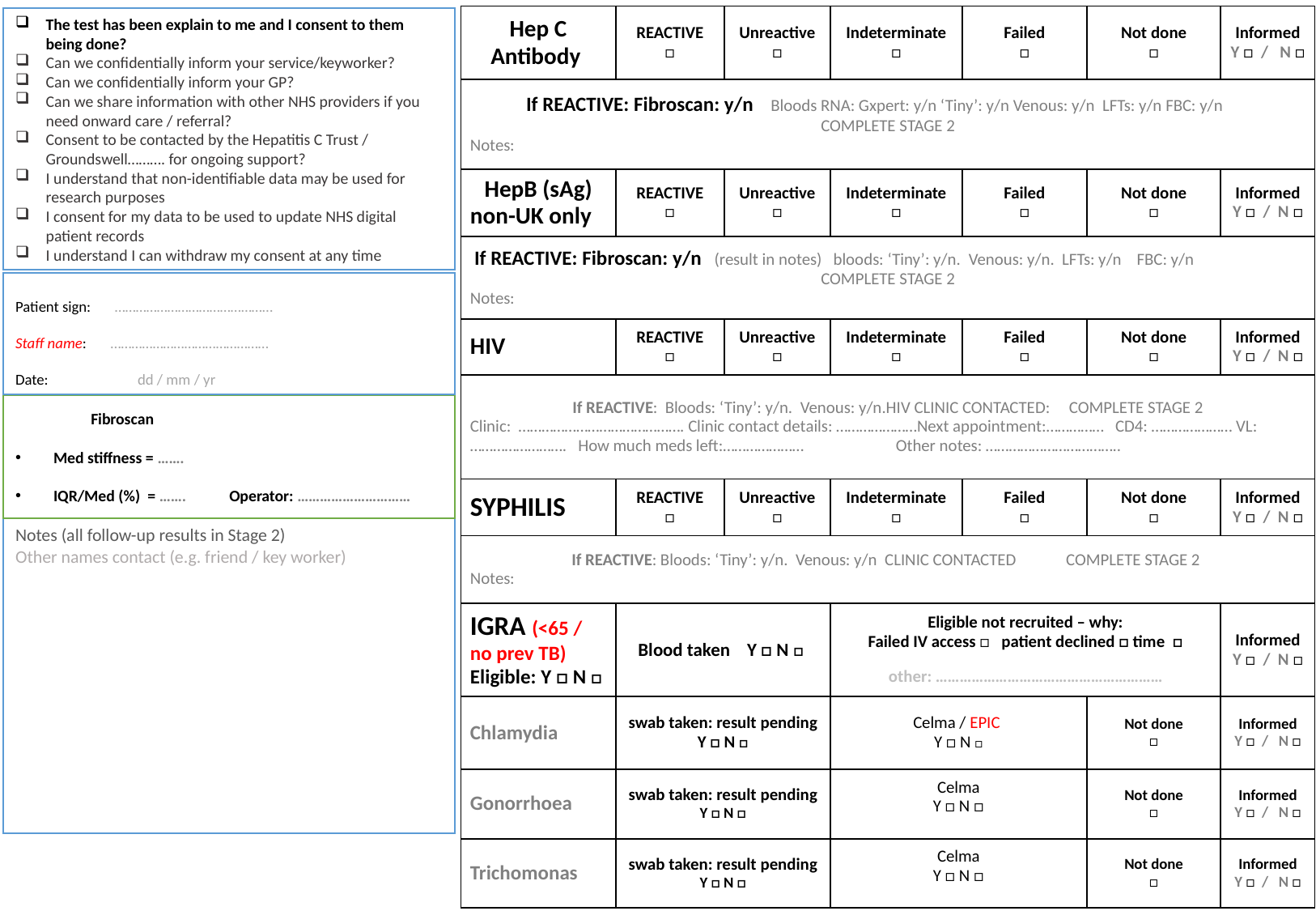

| Hep C Antibody | REACTIVE □ | Unreactive □ | Indeterminate □ | Failed □ | Not done □ | Informed Y □ / N □ |
| --- | --- | --- | --- | --- | --- | --- |
| If REACTIVE: Fibroscan: y/n Bloods RNA: Gxpert: y/n ‘Tiny’: y/n Venous: y/n LFTs: y/n FBC: y/n COMPLETE STAGE 2 Notes: | | | | | | |
| HepB (sAg) non-UK only | REACTIVE □ | Unreactive □ | Indeterminate □ | Failed □ | Not done □ | Informed Y □ / N □ |
| If REACTIVE: Fibroscan: y/n (result in notes) bloods: ‘Tiny’: y/n. Venous: y/n. LFTs: y/n FBC: y/n COMPLETE STAGE 2 Notes: | | | | | | |
| HIV | REACTIVE □ | Unreactive □ | Indeterminate □ | Failed □ | Not done □ | Informed Y □ / N □ |
| If REACTIVE: Bloods: ‘Tiny’: y/n. Venous: y/n.HIV CLINIC CONTACTED: COMPLETE STAGE 2 Clinic: ……………………………………. Clinic contact details: …………………Next appointment:…………… CD4: ………………… VL:……………………. How much meds left:………………… Other notes: …………………………….. | | | | | | |
| SYPHILIS | REACTIVE □ | Unreactive □ | Indeterminate □ | Failed □ | Not done □ | Informed Y □ / N □ |
| If REACTIVE: Bloods: ‘Tiny’: y/n. Venous: y/n CLINIC CONTACTED COMPLETE STAGE 2 Notes: | | | | | | |
| IGRA (<65 / no prev TB) Eligible: Y □ N □ | Blood taken Y □ N □ | | Eligible not recruited – why: Failed IV access □ patient declined □ time □ other: ………………………………………………… | | | Informed Y □ / N □ |
| Chlamydia | swab taken: result pending Y □ N □ | | Celma / EPIC Y □ N □ | | Not done □ | Informed Y □ / N □ |
| Gonorrhoea | swab taken: result pending Y □ N □ | | Celma Y □ N □ | | Not done □ | Informed Y □ / N □ |
| Trichomonas | swab taken: result pending Y □ N □ | | Celma Y □ N □ | | Not done □ | Informed Y □ / N □ |
The test has been explain to me and I consent to them being done?
Can we confidentially inform your service/keyworker?
Can we confidentially inform your GP?
Can we share information with other NHS providers if you need onward care / referral?
Consent to be contacted by the Hepatitis C Trust / Groundswell………. for ongoing support?
I understand that non-identifiable data may be used for research purposes
I consent for my data to be used to update NHS digital patient records
I understand I can withdraw my consent at any time
Patient sign: ………………………………………
Staff name: ………………………………………
Date: dd / mm / yr
Fibroscan
Med stiffness = …….
IQR/Med (%) = …….
Operator: …………………………
Notes (all follow-up results in Stage 2)
Other names contact (e.g. friend / key worker)
